# The metabolite transporters of C_4_ photosynthesis

**DOI:** 10.1093/plcell/koaf019

**Published:** 2025-01-27

**Authors:** Oliver Mattinson, Steven Kelly

**Affiliations:** Department of Biology, University of Oxford, Oxford OX1 3RB, UK; Department of Biology, University of Oxford, Oxford OX1 3RB, UK

## Abstract

C_4_ photosynthesis is a highly efficient form of photosynthesis that utilizes a biochemical pump to concentrate CO_2_ around rubisco. Although variation in the implementation of this biochemical pump exists between species, each variant of the C_4_ pathway is critically dependent on metabolite transport between organelles and between cells. Here we review our understanding of metabolite transport in C_4_ photosynthesis. We discuss how the majority of our knowledge of the metabolite transporters co-opted for use in C_4_ photosynthesis has been obtained from studying C_3_ plants and how there is a pressing need for in planta validation of transporter function in C_4_ species. We further explore the diversity of transport pathways present in disparate C_4_ lineages and highlight the important gaps in our understanding of metabolite transport in C_4_ plants. Finally, through integration of functional and transcriptional data from multiple C_3_ and C_4_ plants, we propose a molecular blueprint for metabolite transport for NAD-malic enzyme C_4_ photosynthesis.

## Introduction

Photosynthesis is the process through which plants use solar energy to convert atmospheric CO_2_ into sugars. Most land plants carry out a form of photosynthesis known as C_3_ photosynthesis, in which rubisco combines CO_2_ with the 5-carbon sugar ribulose 1,5-bisphosphate to generate 2 molecules of 3-phosphoglycerate (3-PGA). However, the efficiency of C_3_ photosynthesis is decreased by a competing reaction wherein rubisco combines O_2_ with ribulose 1,5-bisphosphate. This competing reaction produces 1 molecule each of 3-PGA and 2-phosphoglycolate (2-PG), of which the latter inhibits enzymes of the Calvin-Benson-Bassham Cycle and starch biosynthesis and must be recycled. While the affinity of rubisco for O_2_ is much lower than for CO_2_, atmospheric concentration of O_2_ is 525 times higher than that of CO_2_; thus, the ratio of carboxylation:oxygenation in C_3_ plants is ∼3:1 (Ehleringer et al. 1991). As a consequence, the direct cost of photorespiration and the indirect costs of reassimilation of the released CO_2_ and NH_4_^+^ are thought to result in losses of up to ∼50% of leaf energy (Peterhansel et al. 2010).

To mitigate the cost of photorespiration, several plant lineages have evolved adaptations to increase the concentration of CO_2_ compared with O_2_ around rubisco (Keeley and Rundel 2003; Sage 2016; Edwards 2019; Heyduk et al. 2019). These disparate lineages can be categorized into 2 different photosynthetic types: crassulacean acid metabolism and C_4_ photosynthesis. Both photosynthetic types function by the same principle of initially fixing HCO_3_^−^ instead of CO_2_ using phospho*enol*pyruvate carboxylase (PEPC), an enzyme that is less sensitive to oxygen than rubisco (Sharwood et al. 2016). In C_4_ plants, the separation of initial carbon fixation by PEPC and carbon reduction by rubisco is achieved spatially. CO_2_ is fixed by PEPC in mesophyll cells into 4-carbon organic acids. These 4-carbon organic acids are then moved to a spatially separated location that contains rubisco, where the fixed CO_2_ is then released. While some plants achieve this spatial separation within a single cell (Edwards et al. 2004), in most cases the C_4_ pathway is split between 2 specialized cell types, known as the mesophyll and bundle sheath cells. Irrespective of the anatomical manner in which the partitioning is achieved, the biochemical pump acts to deliver superatmospheric concentrations of CO_2_ to rubisco (Furbank and Hatch 1987). Accordingly, C_4_ plants have decreased rates of photorespiration and increased photosynthetic efficiency and can have increased maximal photosynthetic rates (Evans and von Caemmerer 2000). As a corollary of these adaptations, C_4_ photosynthesis is also associated with enhanced nitrogen use and water use efficiencies, as less nitrogen needs to be invested in rubisco to achieve the same rate of carbon fixation (Sage et al. 1987), and the carbon-concentrating effect allows photosynthetic rate to remain high despite low intercellular CO_2_ concentration caused by stomatal closure (Way et al. 2014).

**Figure 1.**
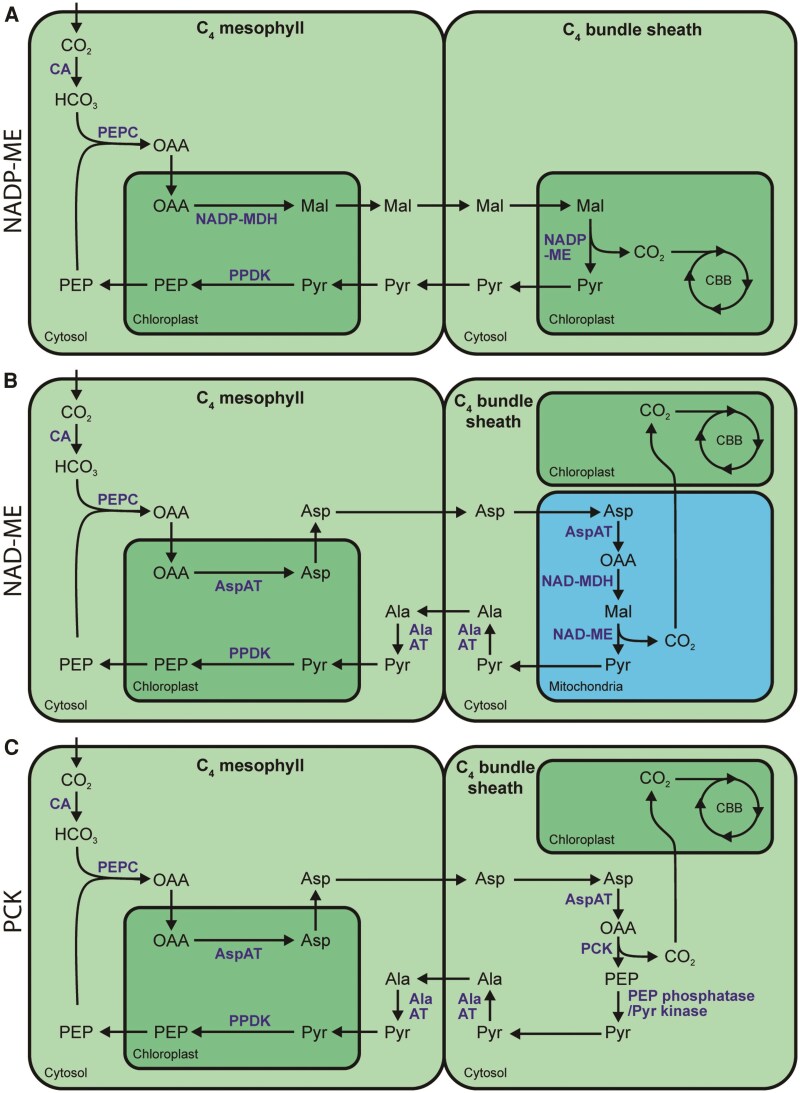
C_4_ photosynthesis subtypes. Diagrams showing the core pathway of **(A)** NADP-ME subtype, **(B)** NAD-ME subtype, and **(C)** PCK subtype C_4_ photosynthesis. Metabolites are shown in black. Enzymes are shown in blue. Abbreviations: Ala, alanine; AlaAT, alanine amino transferase; Asp, aspartate; CA, carbonic anhydrase; CBB, Calvin-Benson-Bassham cycle; Mal, malate; MDH, NADP-malate dehydrogenase; PPDK, pyruvate, phosphate dikinase; Pyr, pyruvate.

C_4_ photosynthesis has evolved independently at least 60 times during the evolution of land plants (Sage 2016). As a consequence of the large number of independent origins, the C_4_ trait encompasses significant variation between species. Biochemically, C_4_ plants are classified into 3 subtypes, based on the decarboxylation enzyme with the highest activity in the leaf (Hatch et al. 1975). These are chloroplastic NADP-malic enzyme (NADP-ME), mitochondrial NAD-malic enzyme (NAD-ME), or cytosolic phospho*enol*pyruvate carboxykinase (PCK) (Fig. 1). While the specialization of the bundle sheath is substantially different between subtypes, all share similar metabolic adaptations in the mesophyll (Fig. 1). The commonalities are: 1) pyruvate is converted to phospho*enol*pyruvate (PEP) by chloroplastic pyruvate, phosphate dikinase. 2) Carbonic anhydrase converts CO_2_ into HCO_3_^−^, and 3) PEPC generates oxaloacetate (OAA) from the combination of HCO_3_^−^ and PEP. As OAA is short-lived and spontaneously decomposes back to pyruvate and CO_2_ (Tsai 1967), OAA is imported into the mesophyll chloroplast, where it is stabilized as either malate [by NADP-malate dehydrogenase (NADP-MDH)] or aspartate [by aspartate aminotransferase (AspAT)], depending on the C_4_ subtype. If OAA is stabilized as malate, then it is decarboxylated in the bundle sheath to produce pyruvate and CO_2_ by chloroplastic NADP-ME (Fig. 1A). If OAA is stabilized as aspartate, then there are 2 potential routes depending on the primary decarboxylase that is used. In NAD-ME subtype C_4_ photosynthesis, the aspartate is imported into the bundle sheath cell mitochondrion, converted back to OAA by mitochondrial AspAT, restabilized as malate by mitochondrial NAD-MDH, and decarboxylated to produce pyruvate and CO_2_ by mitochondrial NAD-ME (Fig. 1B). In PCK subtype C_4_ photosynthesis, the aspartate is converted to OAA by cytosolic aspartate amino transferase and then directly decarboxylated to produce PEP and CO_2_ by cytosolic PCK (Fig. 1C). Although 3 separate subtypes (NADP-ME, NAD-ME, and PCK) have been described, a strict categorization into these subtypes is now considered to be an oversimplification. Instead, many C_4_ plants possess mixed cycles utilizing more than 1 decarboxylase (Furbank 2011; Wang et al. 2014), and aspartate can be used as an alternative transfer metabolite in NADP-ME subtype C_4_ photosynthesis (Leegood and von Caemmerer 1989). Thus, there is likely a wide range in complexity and diversity in the manifestation of C_4_ pathways within and between C_4_ species.

### C_4_ photosynthesis is highly dependent on intracellular metabolite transport

**Figure 2.**
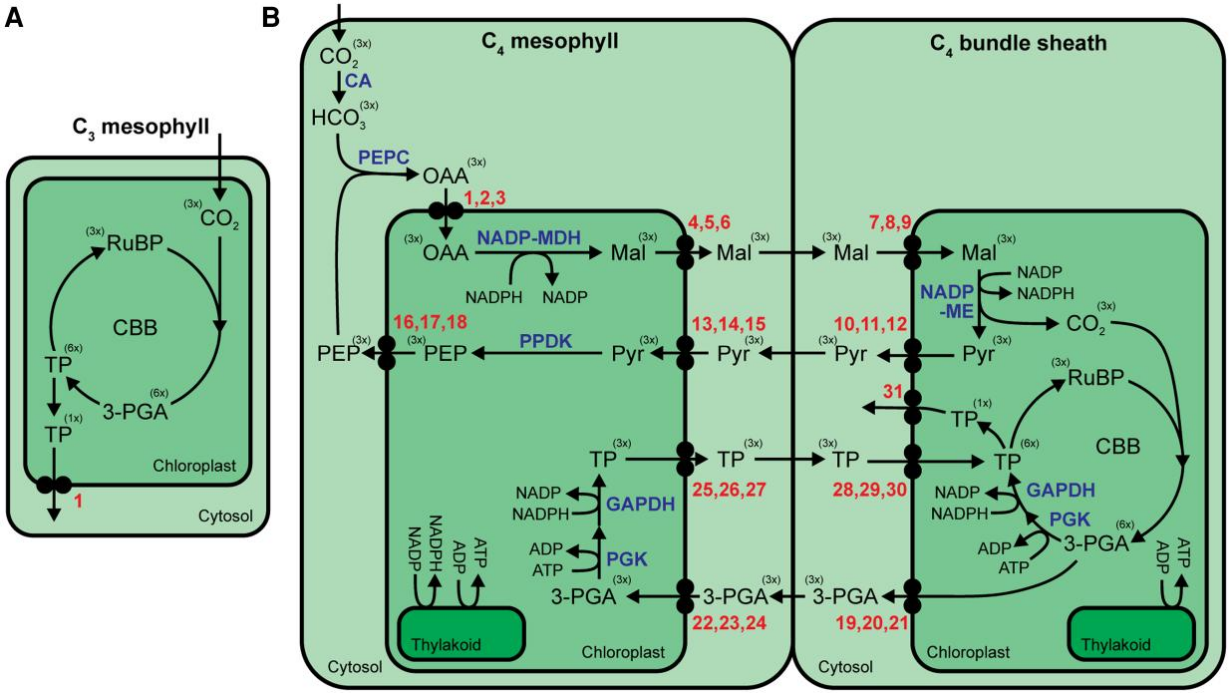
Intracellular metabolite transport in C_3_ and C_4_ photosynthesis. Diagrams showing minimal intracellular metabolite transport required for **(A)** C_3_ photosynthesis, and **(B)** NADP-ME subtype C_4_ photosynthesis. Metabolites are shown in black. Enzymes are shown in blue. Brackets show relative stoichiometries of each metabolite for the production and export of 1 molecule of triose phosphate. Counts of number of transport reactions required for the production and export of 1 molecule of triose phosphate are shown in red. Abbreviations: TP, triose phosphate. For other abbreviations see Fig. 1.

A defining feature of C_4_ photosynthesis is the requirement for extensive movement of metabolites across the membranes of both chloroplasts and mitochondria in multiple different cell types (Fig. 1). Due to this spatial compartmentalization, C_4_ photosynthesis is much more dependent on metabolite transport than C_3_ photosynthesis. In C_3_ photosynthesis the assimilation of 3 molecules of CO_2_ into a single molecule of triose phosphate requires just 1 intracellular metabolite transport step: the export of that triose phosphate from the chloroplast into the cytosol via the triose phosphate translocator (TPT) (Fig. 2A). In contrast, the assimilation of the same number of molecules of CO_2_ using the NADP-ME C_4_ pathway requires at least 31 metabolite transport steps (Weber and von Caemmerer 2010) (Fig. 2B). Despite the importance of intracellular metabolite transport for C_4_ photosynthesis, our understanding of the transporters responsible for these transport steps lags behind our knowledge of the enzymes involved in the C_4_ cycle. This limits our understanding of how C_4_ plants achieve their high photosynthetic efficiency as well as enhanced nitrogen and water use efficiencies. Moreover, this knowledge gap prevents the engineering of C_4_ photosynthesis into C_3_ plants to improve their yield. Here we review the current state of understanding of metabolite transport in C_4_ photosynthesis. As all extant examples of C_4_ photosynthesis evolved from C_3_ photosynthetic ancestors, we discuss how these C_4_ cycle transporters have been co-opted from ancestral functions in C_3_ metabolism. We also highlight the major gaps in our understanding of metabolite transport in C_4_ photosynthesis and, through synthesis of the available transport and transcriptomic data, propose a complete transport model for NAD-ME C_4_ photosynthesis.

### Chloroplast dicarboxylate transport in the C_4_ cycle—co-option of transporters from nitrogen assimilation and redox homeostasis

All known manifestations of C_4_ photosynthesis require dicarboxylate transport across the mesophyll chloroplast envelope, and the NADP-ME subtype also requires dicarboxylate transport across the bundle sheath chloroplast envelope (Fig. 1). Transport of dicarboxylates across the chloroplast envelope occurs primarily via transporters belonging to the divalent anion-sodium symporter family (Sauer et al. 2020). Although this family contains both symporters and exchangers, it is named after the Na^+^:dicarboxylate symport mechanism exhibited by most of its bacterial and mammalian members (Taniguchi and Miyake 2012). To date, all characterized members of the dicarboxylate transporter (DCT or DiT) subfamily from plants function as chloroplast-localized dicarboxylate exchangers and were first identified in pursuit of transporters involved in chloroplast nitrogen metabolism (Taniguchi et al. 2002, 2004; Renné et al. 2003).

**Figure 3.**
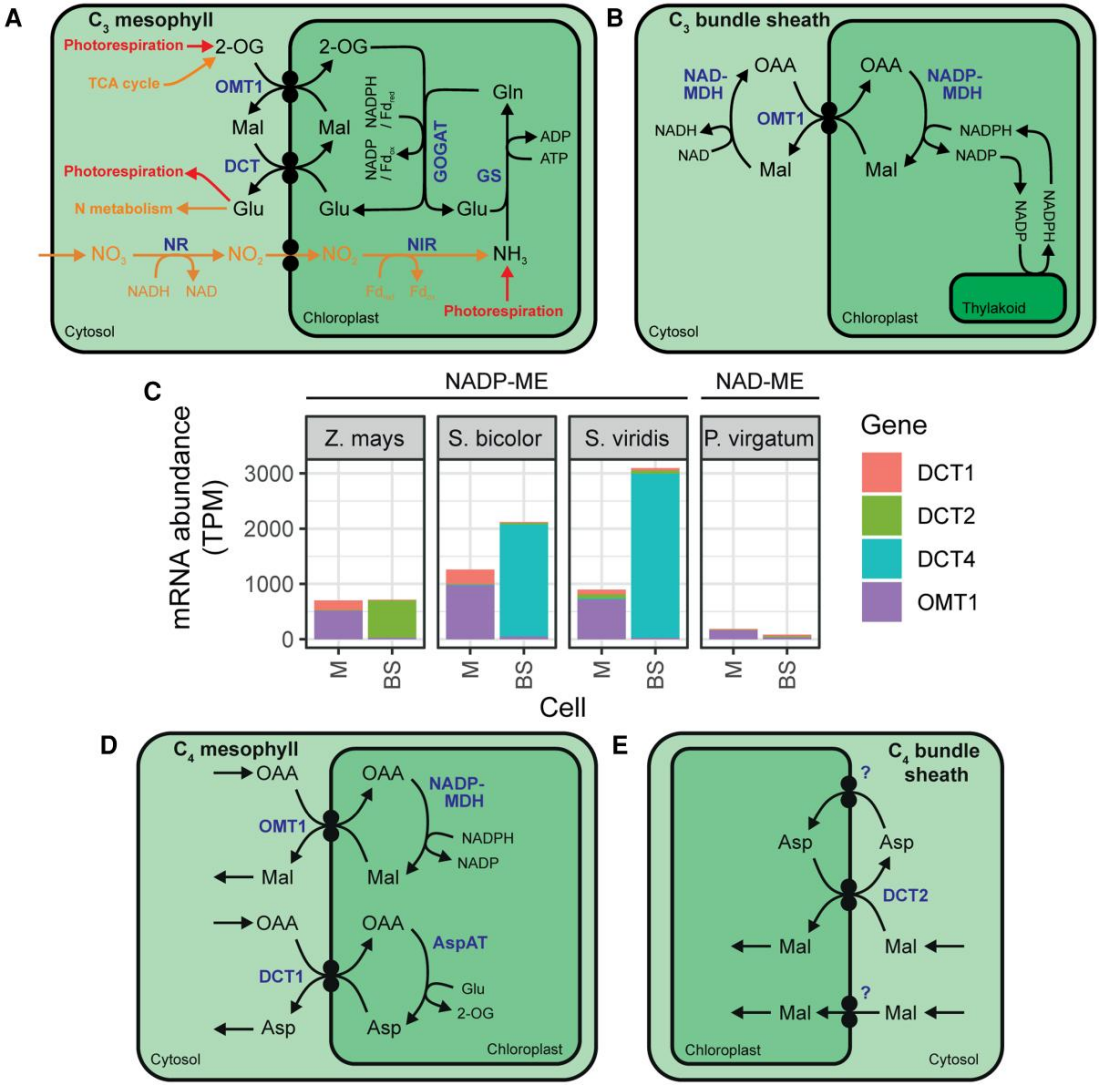
The role of DCT transporters in C_3_ and C_4_ plants. **A)** DCT function in nitrogen assimilation in C_3_ plants. Entry and exit points to primary N assimilation are shown in orange and to secondary (photorespiratory) N assimilation in red. **B)** OMT1 function in redox balancing in C_3_ plants. **C)** Cell-type specific transcript abundance of DCT family genes among NADP-ME and NAD-ME subtype C_4_ monocots. *Z. mays* data from Chang et al. (2012). *S. bicolor* data from Emms et al. (2016). *S. viridis* data from John et al. (2014). *P. virgatum* data from Rao et al. (2016). **D)** Function of OMT1 and DCT1 in mesophyll cell of C_4_ plants. **E)** Hypothesized function of DCT2 in bundle sheath cell of C_4_ plants. Metabolites are shown in black. Enzymes and transporters are shown in blue. Abbreviations: BS, bundle sheath; GOGAT, glutamine oxoglutarate aminotransferase; GS, glutamine synthase; M, mesophyll; NIR, nitrite reductase; NR, nitrate reductase; TPM, transcripts per million. For other abbreviations see Fig. 1.

Photorespiratory nitrogen assimilation via the glutamine synthetase/glutamine oxoglutarate aminotransferase pathway in C_3_ plants requires the exchange of 2-oxoglutarate (2-OG) for glutamate at the chloroplast envelope (Woo et al. 1987). The first characterization of this activity came from the observation of the release of preloaded radio-labeled dicarboxylates from isolated spinach chloroplasts in exchange for external dicarboxylates (Heldt and Rapley 1970). Determination of the transport kinetics for a range of different dicarboxylates suggested the existence of 2 separate exchangers of overlapping specificity: one that preferentially transports aspartate, and one that preferentially transports non-amino acid dicarboxylates (Fig. 3A) (Lehner and Heldt 1978; Anderson and Walker 1983; Dry and Wiskich 1983; Woo et al. 1987). The purification and kinetic characterization of a 2-OG/Malate transporter (OMT1, sometimes referred to as DiT1), which is unable to transport amino acids (Menzlaff and Flügge 1993; Weber et al. 1995; Taniguchi et al. 2002; Renné et al. 2003), matched the first of these hypothesized transporters. Subsequently, a general Dicarboxylate Transporter (DCT, sometimes referred to as DiT2), identified through kinetic analysis of spinach chloroplasts preloaded with glutamate, matched the second (Flügge et al. 1988; Taniguchi et al. 2002; Renné et al. 2003).

Nitrogen assimilation is not the only cellular process that requires dicarboxylate exchange across the chloroplast envelope. Reducing equivalents generated by photosynthetic electron transport must also be exported from the chloroplast to be used by the rest of the cell. One mechanism for this transport is the chloroplast “malate valve” in which an OAA/malate exchanger acts in combination with malate dehydrogenases acting in opposite directions on either side of the membrane (Scheibe 2004) (Fig. 3B). A significantly increased K_m_ for OAA uptake into chloroplasts isolated from *omt1* mutant *A. thaliana* suggests that OMT1 is the high-affinity OAA transporter that forms part of the malate valve in *A. thaliana* chloroplasts (Kinoshita et al. 2011).

In C_4_ photosynthesis, the DCT transporters described above have been co-opted from their ancestral roles to facilitate carbon flux through the C_4_ cycle by mediating dicarboxylate exchange across the chloroplast envelope (Taniguchi et al. 2004). In maize there are 3 *DCT* subfamily genes: *OMT1*, *DCT1*, and *DCT2*. *ZmOMT1* is expressed strongly in the mesophyll, *ZmDCT1* is expressed to a lower level and preferentially in the mesophyll, and *ZmDCT2* is expressed strongly in the bundle sheath (Taniguchi et al. 2004; Majeran et al. 2008; Chang et al. 2012) (Fig. 3C). It has therefore been proposed that ZmOMT1 facilitates exchange of cytosolic OAA with chloroplastic malate in the mesophyll cell as part of the C_4_ cycle (Taniguchi et al. 2004) (Fig. 3D). In a similar manner, ZmDCT1 localized in the mesophyll chloroplast envelope may facilitate the exchange of OAA with chloroplastic aspartate (generated by AspAT) as part of the auxiliary PCK cycle in maize (Fig. 3D). Although the properties of these transporters in maize match the required functions (Taniguchi et al. 2004), and the patterns of expression observed in C_4_ plants indicate they are in the right cell types (Fig. 3C), these hypotheses have not formally been tested; thus, the generation and characterization of *omt1* and *dct1* mutants in C_4_ plants are necessary to confirm these roles.

In contrast to the proposed role of OMT1 in the mesophyll cell chloroplast, ZmDCT2 is proposed to be involved in the import of malate into the bundle sheath chloroplast in the NADP-ME C_4_ cycle (Weissmann et al. 2016) (Fig. 3E). This is supported by the analysis of *dct2* mutant maize, from which isolated bundle sheath chloroplasts show lower rates of malate uptake compared with those from wild-type plants (Weissmann et al. 2016). Additionally, radio-labeled CO_2_ and malate feeding experiments (using whole plants and isolated bundle sheath cells, respectively) indicate an increased production of aspartate in place of malate, consistent with a diversion in flux from the disrupted NADP-ME C_4_ cycle to the auxiliary PCK cycle (Weissmann et al. 2016). It should be noted that DCT2, being an obligate dicarboxylate exchanger, cannot by itself facilitate the net import of malate into the bundle sheath chloroplast that is required for flux through the C_4_ cycle. Therefore, a model has been proposed that involves the existence of a separate, as-yet-unidentified aspartate uniporter (or cation symporter), which imports aspartate into the bundle sheath chloroplast so that DCT2 can carry out aspartate export in exchange for malate import (Weissmann et al. 2016) (Fig. 3E). This proposal is consistent with previous observations that malate-dependent pyruvate formation from maize bundle sheath cells or chloroplasts is stimulated by aspartate (Chapman and Hatch 1979; Jenkins and Boag 1985). Also consistent with the role of DCT transporters in bundle sheath chloroplast malate uptake is the low expression of genes encoding these transporters in species that do not take up malate into bundle sheath chloroplasts, such as *Panicum virgatum* (Fig. 3C), *Gynandropsis gynandra* (Bräutigam et al. 2011), and *Urochloa fusca* (Rao et al. 2016). Therefore, although DCT2 is likely part of the solution, the molecular mechanism of how malate enters the bundle sheath chloroplast in NADP-ME C_4_ photosynthesis is still to be resolved.

### Chloroplast triose phosphate transport in the C_4_ cycle: co-option of a C_3_ redox valve to balance energy metabolism between bundle sheath and mesophyll cells

NADP-ME subtype C_4_ photosynthesis requires the exchange of TP and 3-PGA across the envelope of both mesophyll and bundle sheath chloroplasts (Fig. 2B). This exchange, while not part of the core C_4_ cycle, is a necessary feature of NADP-ME C_4_ photosynthesis due to the downregulation of PSII in the bundle sheath (Woo et al. 1970). This downregulation leads to increased cyclic electron transport around PSI, which produces ATP but not NADPH. The absence of NADPH production assists in kinetically favoring the forward reaction of NADP-ME (Bräutigam et al. 2018). However, the reduction stage of the Calvin-Benson-Bassham cycle also located in the bundle sheath cell requires NADPH, and stoichiometrically only one-half of this can be provided by NADP-ME through decarboxylation of malate (Weber and von Caemmerer 2010). Therefore, one-half of the 3-PGA generated in the bundle sheath chloroplast must be transported to the mesophyll chloroplast to be converted to triose phosphate, through the actions of phosphoglycerate kinase (PGK) and glyceraldehyde 3-phosphate dehygrogenase (GAPDH), utilizing NADPH produced by linear electron transport in the mesophyll (Taniguchi and Miyake 2012). Triose phosphate must then be returned to the bundle sheath chloroplast for the completion of the Calvin-Benson-Bassham cycle (Weber and von Caemmerer 2010).

**Figure 4.**
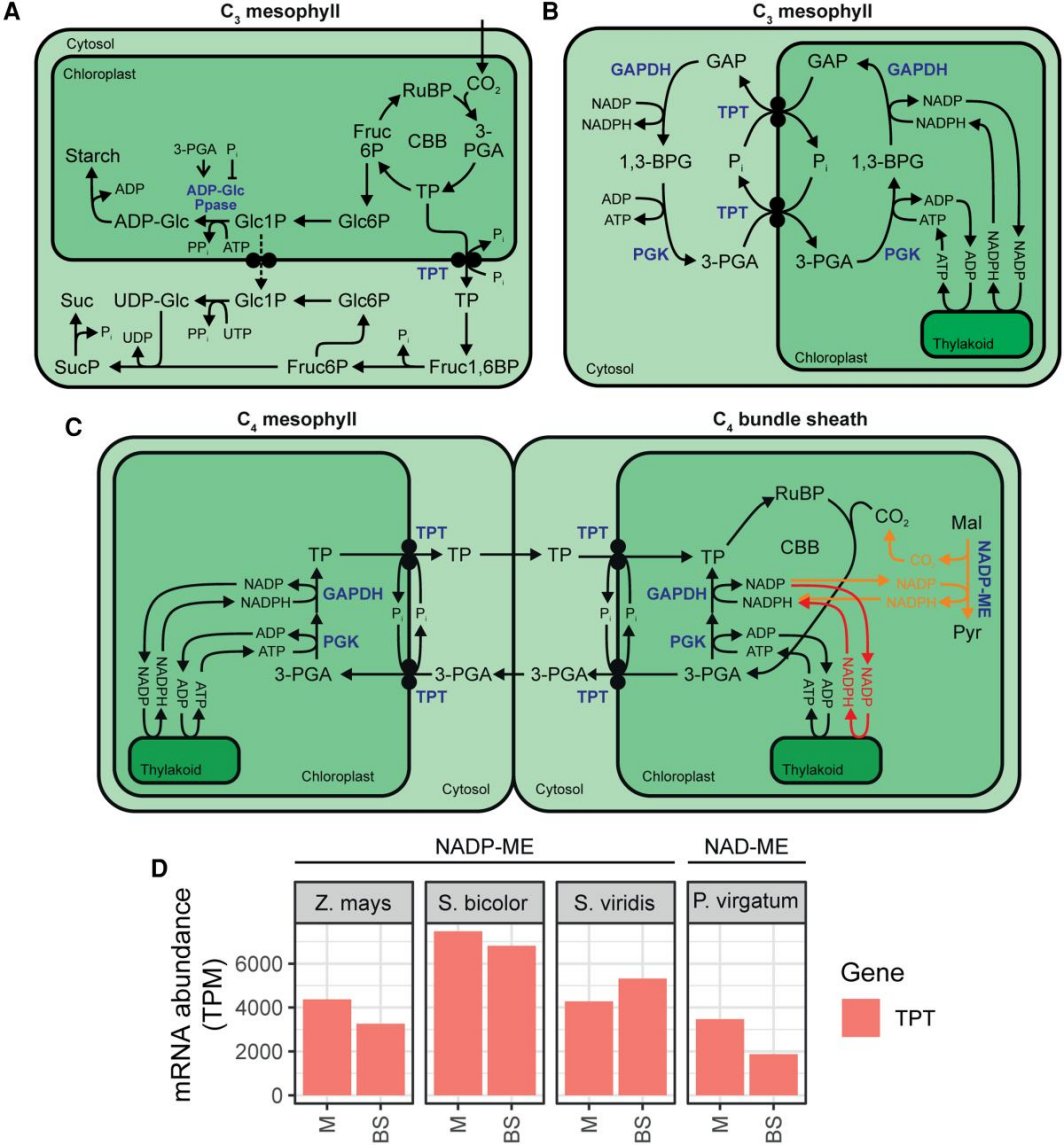
The role of TPT in C_3_ and C_4_ plants. **A)** TPT function in photosynthetic TP export and coordination of starch and sucrose biosynthesis in C_3_ plants. **B)** TPT function in exporting reducing equivalents and ATP from the chloroplast in C_3_ plants. **C)** Functions of TPT in C_4_ plants. NADP-ME subtype-only parts of pathway are shown in orange. NAD-ME subtype-only parts of pathway are shown in red. **D)** Cell-type specific transcript abundance of TPT genes among NADP-ME and NAD-ME subtype C_4_ monocots. Data sources described in Fig. 3. Metabolites are shown in black. Enzymes and transporters are shown in blue. Abbreviations: 1,3BPG, 1,3-bisphosphoglycerate; ADP-Glc, ADP-glucose; BS, bundle sheath; Fruc1,6BP, fructose 1,6-bisphosphate; Fruc6P, fructose 6-phosphate; GAP, glyceraldehyde 3-phosphate; Glc1P, glucose 1-phophate; Glc6P, glucose-6-phophate; M, mesophyll; TPM, transcripts per million; UDP-Glc, UDP-glucose. For other abbreviations see previous figures.

The primary transport flux of triose phosphates across the chloroplast inner envelope occurs via the TPT. TPT is a member of the larger Drug/Metabolite Transporter superfamily. TPT family proteins are specific to eukaryotes (Jack et al. 2001) and catalyze the exchange of phosphorylated monocarboxylates with inorganic phosphate (P_i_) (Lee et al. 2017). The first observation of TPT activity was the exchange of P_i_ with phosphorylated 3-carbon compounds across the envelopes of isolated spinach chloroplasts (Heldt and Rapley 1970). The rate of exchange was high with triose phosphates phosphorylated on the third carbon, lower for those phosphorylated on the second carbon, and lower still for sugar phosphates (Heldt and Rapley 1970; Fliege et al. 1978). Given these transport properties, an obvious function for TPT is the export of the primary products of photosynthesis from the chloroplast to the cytosol. Accordingly, in C_3_ plants TPT plays a direct role in the coordination between sucrose and starch biosynthesis (Riesmeier et al. 1993) (Fig. 4A).

In addition to the main role of TPT above, supplementary roles for TPT in C_3_ plants have also been described. Similar to the chloroplast malate valve, TPT can act in concert with chloroplastic and cytosolic enzymes to indirectly export reducing equivalents to the cytosol, although in this case TPT also results in the indirect export of ATP to the cytosol (Taniguchi and Miyake 2012). Here, in the chloroplast 3-PGA is converted by the ATP-consuming PGK and NADPH-consuming GAPDH into GAP, while in the cytosol GAP is converted by the NADH-producing GAPDH and ATP-producing PGK back to 3-PGA. In this way, both ATP and reducing power are indirectly “shuttled” from the chloroplast to the cytosol (Fig. 4B). In C_4_ plants, it is this shuttle-like function of TPT that is co-opted into the C_4_ cycle. However, unlike in C_3_ plants where this shuttle functions in the context of a single cell (Fig. 4B), in C_4_ plants it is distributed across the 2 cell types to transfer reducing equivalents produced by linear electron transport in mesophyll chloroplasts to bundle sheath chloroplasts (Fig. 4C). Consistent with this role, NADP-ME subtype C_4_ plants show higher leaf *TPT* RNA and TPT protein levels than C_3_ plants (Bräutigam et al. 2008; Gowik et al. 2011), and high *TPT* RNA and TPT protein levels in both mesophyll and bundle sheath (Chang et al. 2012; John et al. 2014; Emms et al. 2016) (Fig. 4D).

Although this shuttle is thought to primarily function in NADP-ME C_4_ photosynthesis, it should be noted that *TPT* is also expressed to high levels in both mesophyll and bundle sheath cells of NAD-ME subtype C_4_ plants such as *P. virgatum* (Fig. 4D) and *G. gynandra* (Rao et al. 2016), despite the fact that NAD-ME plants do not show depleted bundle sheath chloroplast PSII activity (Koteyeva et al. 2014). It is therefore possible that redox/ATP balancing is required between mesophyll and bundle sheath chloroplasts in all C_4_ plants, not just in NADP-ME subtype C_4_ plants where the downregulation of PSII in the bundle sheath makes this need more apparent.

### Chloroplast pyruvate import in the C_4_ cycle: co-option of the entry point transporter for terpenoid biosynthesis

All known C_4_ cycles require pyruvate import across the mesophyll chloroplast envelope (Fig. 2). Unlike all other transport reactions discussed in this review, in which the transport reaction was first demonstrated in C_3_ plants, pyruvate uptake into chloroplasts was first demonstrated using chloroplasts isolated from the mesophyll cells of the C_4_ plant *Digitaria sanguinalis* (Huber and Edwards 1977). It was later shown that this transport reaction in maize is light dependent (Flügge et al. 1985), and in *Panicum miliaceum* that pyruvate uptake is directly dependent on the proton gradient that is generated across the chloroplast envelope under illumination (Ohnishi and Kanai 1987). Subsequent sampling across a broader species range revealed that light-dependent chloroplast pyruvate uptake can be divided into 2 mechanisms: H^+^- and Na^+^-dependent (Aoki et al. 1992). H^+^-dependent pyruvate uptake is limited to 2 sister lineages within the grasses that are both derived from the same origin of C_4_ photosynthesis (Grass Phylogeny Working Group II 2012): the Arundinelleae and Andropogoneae, the latter of which contains several important C_4_ crop species such as maize, sugarcane, and sorghum. Na^+^-dependent pyruvate uptake, on the other hand, was found in all other monocot as well as all dicot clades tested so far (Aoki et al. 1992). Thus, there are likely 2 molecularly distinct transporter families that facilitate pyruvate uptake into mesophyll cell chloroplasts.

**Figure 5.**
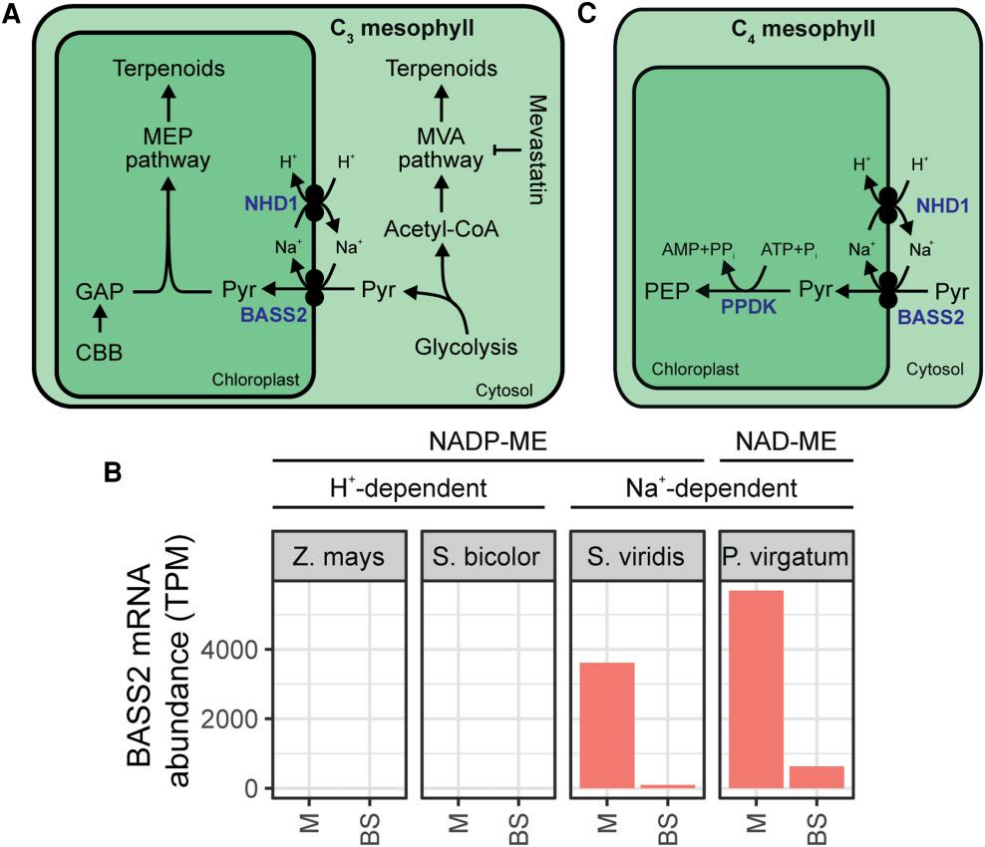
The role of BASS2 in C_3_ and C_4_ plants. **A)** Function of BASS2 in C_3_ plants. **B)** Cell-type specific transcript abundance of BASS2 gene among NADP-ME and NAD-ME subtype C_4_ monocots. H^+^ or Na^+^ dependence of mesophyll cell pyruvate uptake is also indicated. Data sources described in Fig. 3. **C)** Function of BASS2 in C_4_ plants. Metabolites are shown in black. Enzymes and transporters are shown in blue. Abbreviations: BS, bundle sheath; M, mesophyll; MEP, methyl-D-erythritol phosphate; MVA, mevalonate; TPM, transcripts per million. For other abbreviations, see previous figures.

Knockout studies in *A. thaliana* identified that the likely candidate for Na^+^-dependent pyruvate import was BASS2 in conjunction with Na^+^/H^+^ antiporter 1 (NHD1) (Furumoto et al. 2011) (Fig. 5A). Analysis of these plants led to the proposal that BASS2 functions in C_3_ plants to import pyruvate into the chloroplast for entry into the methylerythritol phosphate pathway for terpenoid biosynthesis (Furumoto et al. 2011) (Fig. 5A). Both *BASS2* and *NHD1* are expressed to higher levels in the leaves of Na^+^-type C_4_ species than in close C_3_ relatives (Bräutigam et al. 2011, 2014) but not H^+^-type C_4_ species or C_3_ species (Rao et al. 2016) (Fig. 5B). BASS2 also facilitates pyruvate uptake when heterologously expressed in *E. coli*, which is increased by the addition of Na^+^ (Furumoto et al. 2011). The model for BASS2 function in C_4_ species thus involves the utilization by NHD1 of the light-dependent proton gradient across the chloroplast inner envelope to couple the import of H^+^ down the proton gradient to the export of Na^+^ into the intermembrane space. This enables pyruvate to be imported by pyruvate/Na^+^ symport through BASS2 (Fig. 5C). Although there is strong evidence to support a role for BASS2 in Na^+^-dependent C_4_ photosynthesis, physiological evaluation of loss of function or RNAi lines in C_4_ species has not yet been carried out. Moreover, the identity of the H^+^-dependent pyruvate uptake transporter is yet to be discovered.

### Chloroplast PEP export in the C_4_ cycle: co-option and reversal of the PEP/P_i_ translocator

**Figure 6.**
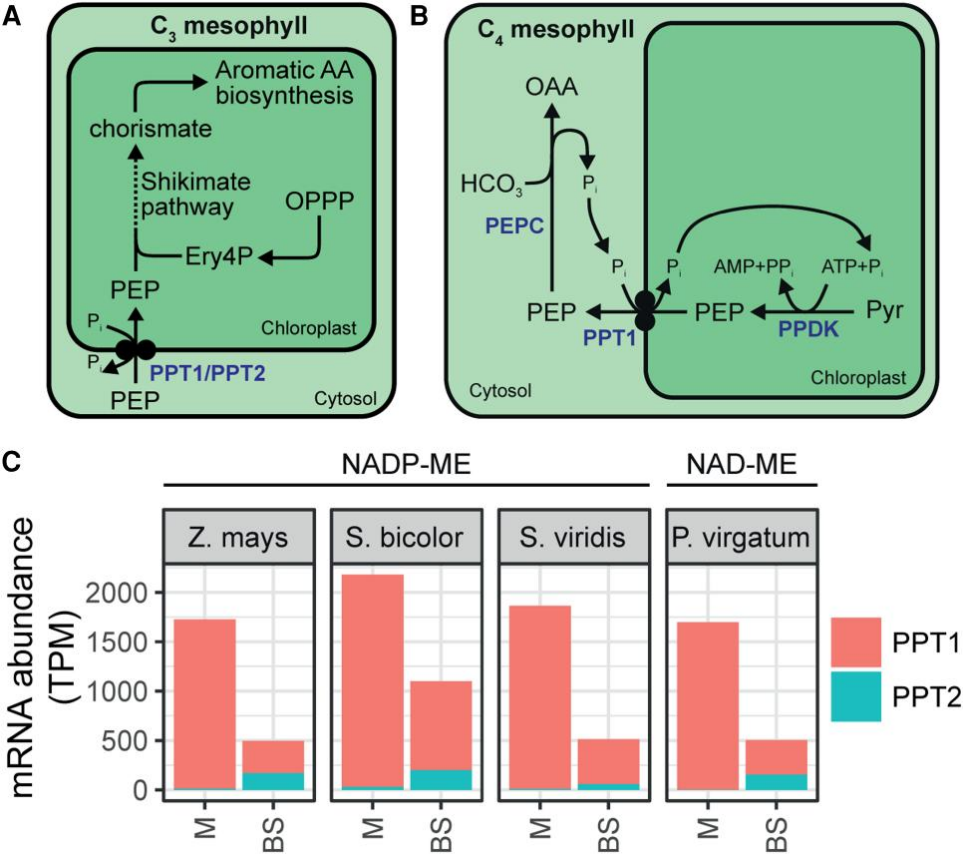
The role of PPT in C_3_ and C_4_ plants. **A)** PPT function in chloroplastic PEP import for the shikimate pathway in C_3_ plants. **B)** Function of PPT in C_4_ plants. **C)** Cell-type specific transcript abundance of PPT genes among NADP-ME and NAD-ME subtype C_4_ monocots. Abbreviations: BS, bundle sheath; M, mesophyll; TPM, transcripts per million. Data sources described in Fig. 3. Metabolites and pathways are shown in black. Transporters are shown in blue. For abbreviations, see Fig. 1.

The final transport step of all known C_4_ cycles is the export of PEP across the chloroplast envelope in the mesophyll cell (Fig. 2). In C_3_ plants, this transport function is carried out by a member of the TPT family of transporters called the PEP/P_i_ translocator (PPT), which transports triose phosphates that have been phosphorylated on the second carbon (Fischer et al. 1997). In *A. thaliana* there are 2 *PPT* genes: *PPT1* and *PPT2*. While *PPT2* is ubiquitously expressed throughout the leaf, *PPT1* is specifically expressed in the vasculature of leaves and roots (Fischer et al. 1997; Knappe et al. 2003). Both PPT1 and PPT2 are thought to import PEP into the chloroplast for entry into the shikimate pathway and production of aromatic amino acids (Knappe et al. 2003; Voll et al. 2003) (Fig. 6A). Loss-of-function plants have impaired chloroplast development caused by a deficit in PEP-derived phenylalanine and downstream phenolic compounds (Voll et al. 2003).

In C_4_ plants of all subtypes, the inverse of the C_3_ plant PEP transport reaction described above is required. Rather than PEP import, PEP must be exported from the mesophyll chloroplast to provide the substrate for PEPC (Fig. 6B). Usually, an ortholog of *PPT1* has been co-opted for function in the C_4_ cycle (Lyu et al. 2020) and correspondingly shows higher RNA and protein levels in C_4_ plants than in C_3_ relatives (Bräutigam et al. 2008, 2011, 2014; Gowik et al. 2011), as well as preferential expression in the mesophyll (Chang et al. 2012; John et al. 2014; Emms et al. 2016; Rao et al. 2016) (Fig. 6C). Although transport activity and cell-type expression data are highly indicative, PPT function in the C_4_ cycle has never been directly tested through analysis of *PPT* knockdown or knockout C_4_ plants.

### Mitochondrial pyruvate transport in the C_4_ cycle: co-option and reversal of the transporter for pyruvate uptake

**Figure 7.**
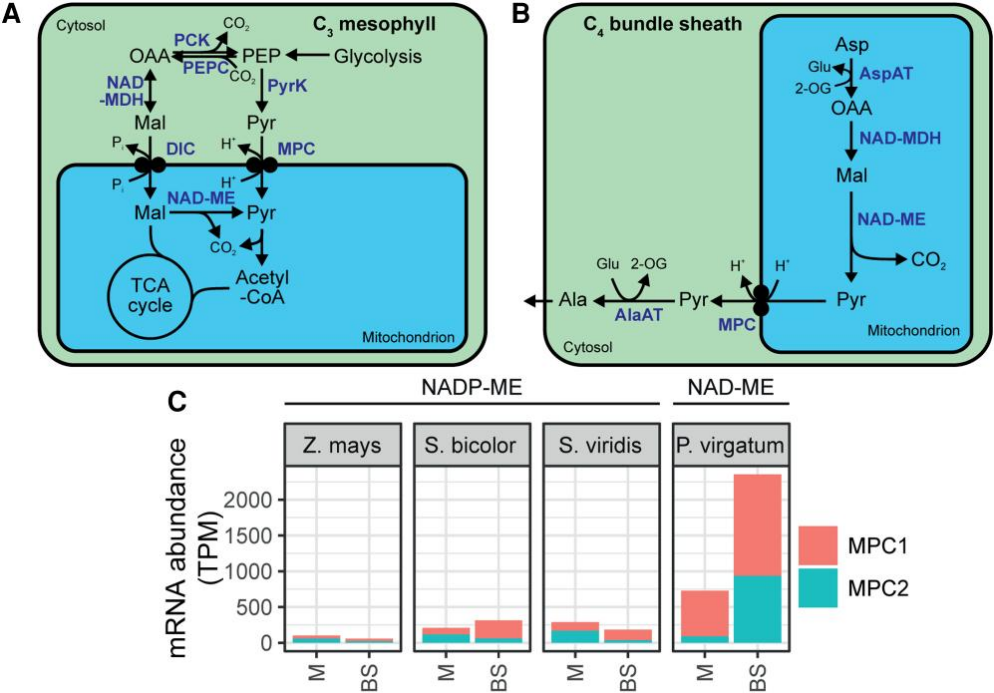
The role of MPC in C_3_ and C_4_ plants. **A)** The function of MPC in C_3_ plants. **B)** The function of MPC in C_3_ plants. **C)** Cell-type specific transcript abundance of MPC genes in NADP-ME and NAD-ME subtype C_4_ monocots. Abbreviations: BS, bundle sheath; M, mesophyll; PyrK, pyruvate kinase; TPM, transcripts per million. Data sources described in Fig. 3. Metabolites and pathways are shown in black. Enzymes and transporters are shown in blue. For other abbreviations, see previous figures.

The NAD-ME subtype C_4_ cycle requires export of pyruvate across the mitochondrial membrane in the bundle sheath cell (Fig. 1B). Across eukaryotes, pyruvate/H^+^ symport into mitochondria is required for anaplerotic provision of pyruvate to the TCA cycle (Fig. 7A) and is facilitated by members of the Mitochondrial Pyruvate Carrier (MPC) family (Halestrap 1975; Herzig et al. 2012). The MPC family in plants comprises multiple members, and mitochondria isolated from *mpc* mutants in *A. thaliana* show significantly decreased pyruvate uptake rates and pyruvate-dependent O_2_ consumption (Le et al. 2021). As for PPT1 described above, the direction of transport of MPC required for function in the NAD-ME C_4_ cycle is opposite to that which has been described in C_3_ plants and other eukaryotes (Fig. 7B). Also as for PPT1, transcriptomic data in C_4_ species supports the putative role of MPC family proteins in carrying out this transport reaction. For example, the NAD-ME subtype monocot *P. virgatum* shows preferential expression in the bundle sheath for *MPC1* and *MPC2* (Rao et al. 2016) (Fig. 7C). This is in contrast to NADP-ME subtype monocots, which show only very low bundle sheath expression of *MPC* genes, as would be expected given the lack of mitochondrial pyruvate export in this subtype (Chang et al. 2012; John et al. 2014; Emms et al. 2016) (Fig. 7C).

Interestingly, similar to pyruvate uptake into chloroplasts described above, there may be multiple potential routes for pyruvate export from mitochondria in NAD-ME C_4_ species. The rationale for this proposal is that mitochondria isolated from *mpc1* knockout *A. thaliana* are capable of exporting large quantities pyruvate (Le et al. 2021), indicating the presence of another, as-yet-unidentified mitochondrial pyruvate exporter. Thus, while it is plausible that MPC may play a role in NAD-ME C_4_ photosynthesis in *P. virgatum*, the precise role that it plays needs to be validated. Moreover, the identity of any uncharacterized pyruvate exporters and their role in NAD-ME C_4_ species remains to be elucidated.

### Mitochondrial malate transport in the C_4_ cycle: co-option of a redox exchange mechanism to support aspartate uptake

**Figure 8.**
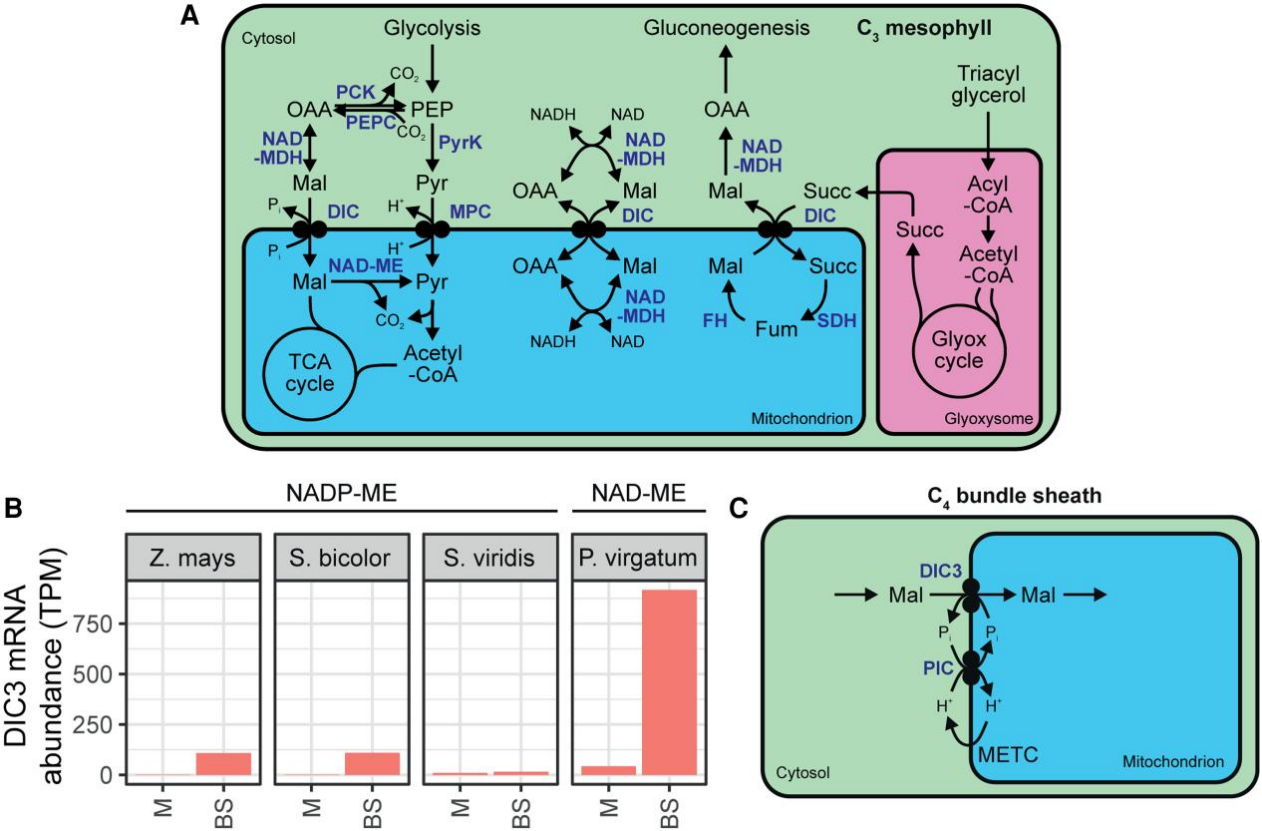
The functions of DIC in C_3_ and C_4_ plants. **A)** Left: Anaplerotic provision of malate to TCA cycle. Centre: Exchange of reducing equivalents between cytosol and mitochondria. Right: Mitochondrial malate/succinate exchange in conjunction with glyoxylate cycle and gluconeogenesis. **B)** Cell type–specific transcript abundance of DIC genes among NADP-ME and NAD-ME subtype C_4_ monocots. Abbreviations: BS, bundle sheath; FH, fumarate hydratase; Glyox cycle, glyoxylate cycle; M, mesophyll; METC, mitochondrial electron transport chain; SDH, succinate dehydrogenase; Succ, succinate; TPM, transcripts per million. Data sources described in Fig. 3. **C)** Function of DIC in C_4_ plants. Metabolites and pathways are shown in black. Enzymes and transporters are shown in blue. For other abbreviations, see previous figures.

The NAD-ME subtype C_4_ cycle requires net import of aspartate into the bundle sheath mitochondria for operation of the CO_2_-concentrating mechanism (Fig. 1B). Mitochondrial aspartate import also requires uptake of 2-OG to support the trans-amination reaction catalyzed by aspartate aminotransferase to convert aspartate into OAA. However, mitochondria isolated from the NAD-ME C_4_ plant *Atriplex spongiosa* were found to require not only 2-OG but also malate and P_i_ to sustain maximal rates of pyruvate and O_2_ formation from aspartate (Kagawa and Hatch 1975; Furbank et al. 1990). The exchange of malate and P_i_ across the mitochondrial membrane is known to be carried out by members of the DIC family of transporters in eukaryotes (Palmieri et al. 2008). In C_3_ plants, these transporters also carry out the counter exchange of several dicarboxylates (including malate, OAA, and succinate), and accordingly several potential roles have been proposed (Fig. 8A). First, DICs may facilitate the anaplerotic provision of dicarboxylates for the TCA cycle (Palmieri et al. 2008), which is consistent with the fact that malate and succinate oxidation in plant mitochondria is inhibited by known inhibitors of DIC (DeSantis et al. 1976; Day and Hanson 1977). Second, DICs may also exchange malate and OAA and act in concert with cytosolic and mitochondrial MDHs to transfer reducing equivalents between cytosol and mitochondria in an analogous way to OMT1 in the chloroplast (Palmieri et al. 2008). Third, high expression of *DIC1* and *DIC2* in cotyledons may indicate a role in the exchange of OAA and malate with succinate across the mitochondrial membrane that is required during the mobilization of storage lipids via the glyoxylate cycle and gluconeogenesis (Palmieri et al. 2008).

Transcriptomic data have supported a role for DIC in the NAD-ME cycle. Among C_4_ monocots, 2 members of the DIC family are expressed in a strongly bundle sheath–preferential manner in the NAD-ME subtype species *P. virgatum* (Rao et al. 2016) but not in NADP-ME subtype species (Chang et al. 2012; John et al. 2014; Emms et al. 2016) (Fig. 8B). In dicots, *DIC1* shows significantly bundle sheath–preferential expression in the NAD-ME subtype C_4_ species *G. gynandra* (Aubry et al. 2014), and this gene is significantly upregulated compared with its close C_3_ relative *G. spinoza* (Bräutigam et al. 2011). The expression patterns above for *DIC* correlate with the expression of genes encoding the mitochondrial Phosphate Carrier, a P_i_/H^+^ symporter (Hamel et al. 2004). Together, this suggests a mechanism by which malate import (and P_i_ export) through DIC is coupled to the proton gradient across the mitochondrial membrane via H^+^ and P_I_ symport through Phosphate Carrier (Fig. 8C).

### Mitochondrial 2-OG transport in the C_4_ cycle: co-option of a multifunctional dicarboxylate exchanger to support aspartate transamination

**Figure 9.**
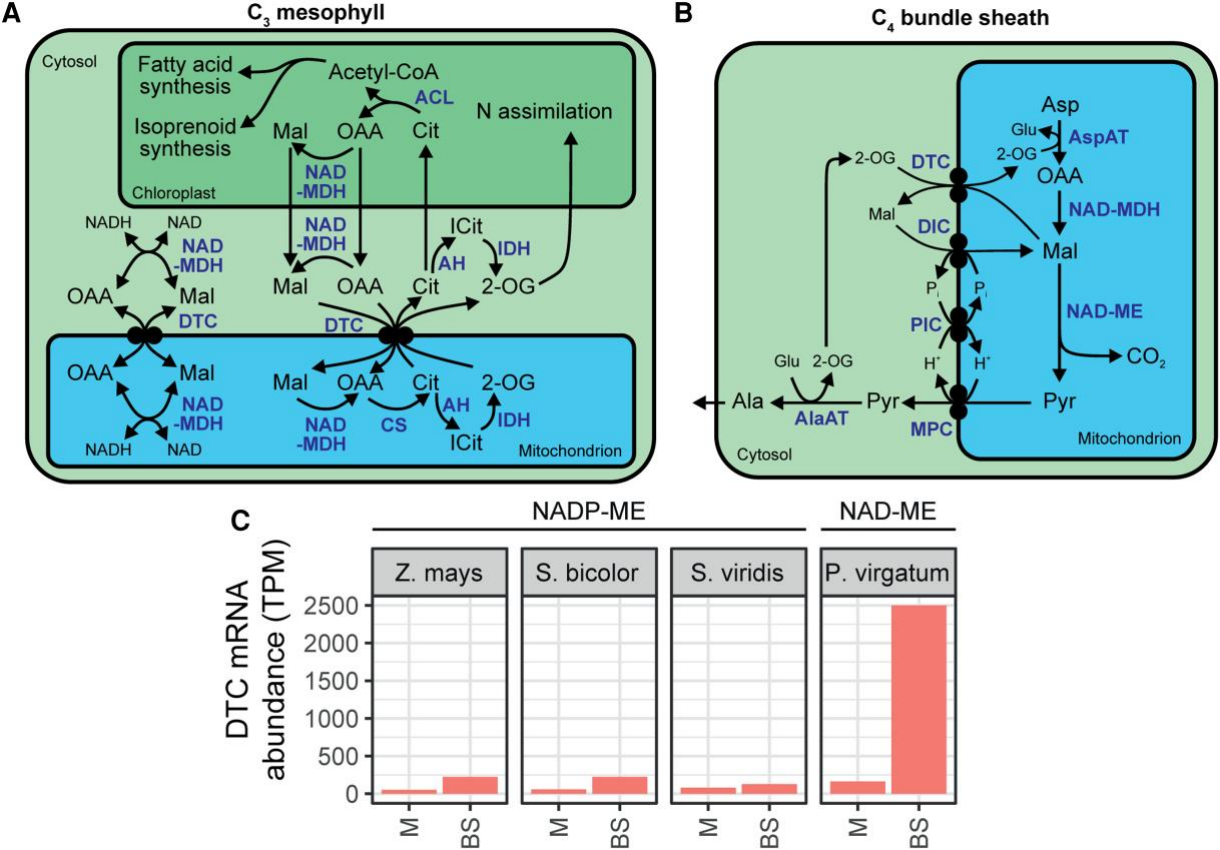
The role of DTC in C_3_ and C_4_ plants. **A)** Possible functions of DTC in C_3_ plants. Left: Exchange of reducing equivalents between cytosol and mitochondria. Right: Exchange of malate and/or oxaloacetate for citrate and 2-OG for chloroplastic fatty acid/isoprenoid synthesis and nitrogen assimilation respectively. **B)** The proposed role of DTC in NAD-ME C_4_ photosynthesis. **C)** Cell-type specific transcript abundance of DTC genes among NADP-ME and NAD-ME subtype C_4_ monocots. Data sources described in Fig. 3. Metabolites and pathways are shown in black. Enzymes and transporters are shown in blue. Abbreviations: 2-OG, 2-oxoglutarate; ACL, ATP citrate lyase; AH, aconitase; BS, bundle sheath; Cit, citrate; CS, citrate synthase; Fum, fumarate; ICit, isocitrate; IDH, isocitrate dehydrogenase; M, mesophyll; TPM, transcripts per million. For other abbreviations, see previous figures.

As discussed above, the net import of aspartate to the mitochondria of the bundle sheath cell also requires the uptake of 2-OG to support the trans-amination reaction catalyzed by aspartate amino transferase. Candidates for this 2-OG uptake are the mitochondrial DTC transporters that catalyze the strict counter-exchange of dicarboxylates (including 2-OG, malate, OAA, succinate) and tricarboxylates (including citrate, isocitrate) (Taniguchi and Sugiyama 1996; Picault et al. 2002). The role for DTC transporters in C_3_ plants has not been well characterized, although multiple hypotheses have been proposed (Fig. 9A). First, the capacity for malate/OAA exchange means a redox valve function is possible (Picault et al. 2002). Second, the capacity for citrate exchange with OAA and/or malate could play a role in fatty acid elongation and isoprenoid synthesis (Picault et al. 2002). Third, DTC, through its capacity to exchange various TCA cycle intermediates, may be involved in the direct export of 2-OG to support nitrogen assimilation via the glutamine synthetase/glutamine oxoglutarate aminotransferase cycle (Picault et al. 2002).

A role for DTC in facilitating mitochondrial 2-OG uptake in NAD-ME subtype C_4_ photosynthesis has been proposed, based on light-dependent, leaf bundle sheath–specific expression, and developmental regulation similar to other C_4_ cycle genes (Taniguchi and Sugiyama 1997; Aubry et al. 2014) (Fig. 9B). This is further supported by transcriptomic data, with *DTC* genes showing high, bundle sheath–specific expression in NAD-ME (Rao et al. 2016) but not in NADP-ME subtype C_4_ monocot species (Chang et al. 2012; John et al. 2014; Emms et al. 2016) (Fig. 9C). Thus, given their expression and known function in C_3_ plants, it is likely that mitochondrial DTC transporters perform the role of supplying 2-OG to support aspartate trans-amination as part of the NAD-ME C_4_ cycle.

### Mitochondrial aspartate transport in the C_4_ cycle: co-option of a photorespiratory redox shuttle

**Figure 10.**
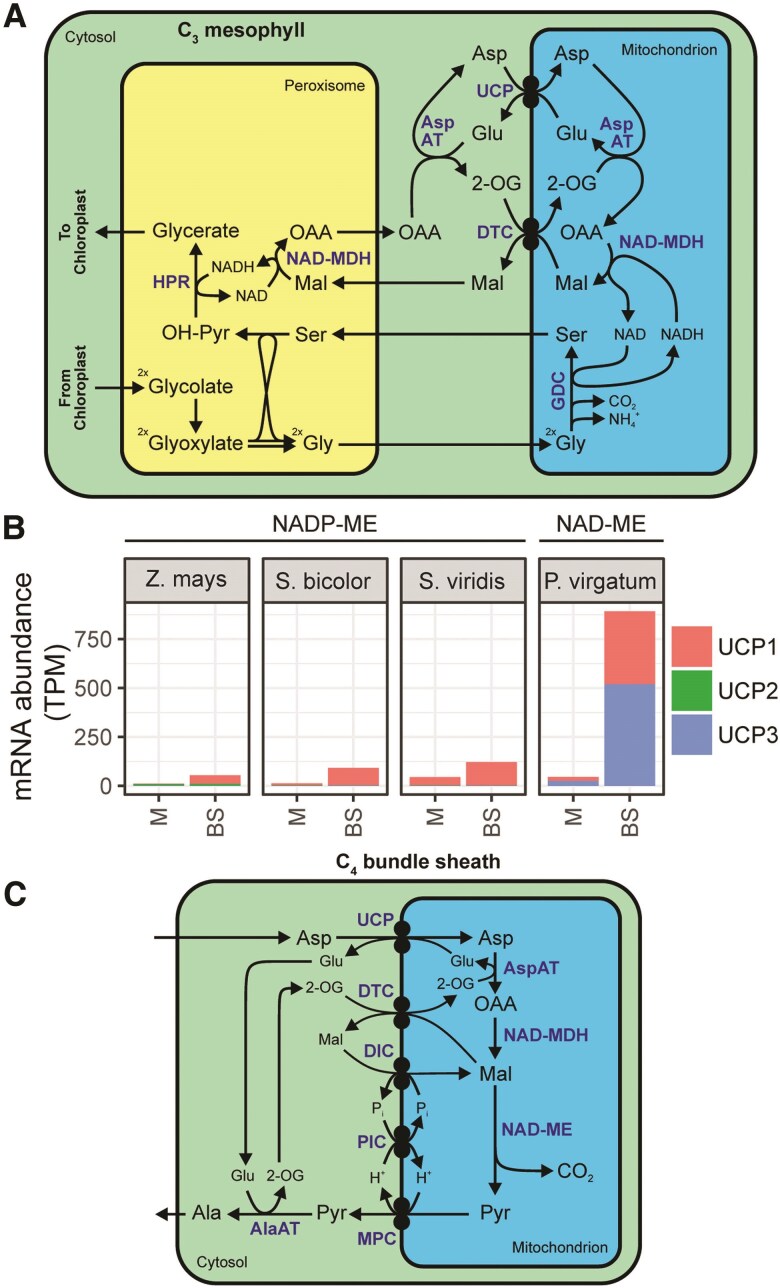
The role of UCP in C_3_ and C_4_ plants. **A)** The role of UCP transporters in respiration and photorespiration in C_3_ plants. **B)** Cell type–specific transcript abundance of UCP genes among NADP-ME and NAD-ME subtype C_4_ monocots. Data sources described in Fig. 3. **C)** A complete molecular blueprint for metabolite transport at the mitochondrial envelope to support NAD-ME subtype C_4_ photosynthesis. Metabolites are shown in black. Enzymes and transporters are shown in blue. Abbreviations: BS, bundle sheath; M, mesophyll; TPM, transcripts per million. For abbreviations, see previous figures.

While the DTC and DIC transporters discussed above are capable of moving dicarboxylates, none have been demonstrated to be capable of moving amino acids nor supporting the net flux of carbon through the NAD-ME subtype C_4_ cycle. Thus, the final piece in the puzzle of mitochondrial metabolite transport in NAD-ME subtype C_4_ photosynthesis is the net uptake of aspartate into mitochondria. Recently it has been shown that Plant Uncoupling Mitochondrial Proteins (PUMPs, also known as UCPs) are able to exchange amino acids (such as aspartate and glutamate), as well as dicarboxylates (such as malate and succinate) and other small compounds (such as sulfate, thiosulfate, and P_i_) (Monné et al. 2018). Through a combination of biochemical/physiological assays and in vitro transport activity measurements, it was suggested that UCPs in C_3_ plants may function as a redox valve to supply reducing equivalents from photorespiratory mitochondrial glycine decarboxylation to support peroxisomal hydroxypyruvate reductase (Monné et al. 2018). This proposed role provides a molecular understanding of a known transfer of reducing equivalents between mitochondria and peroxisomes in which glutamate and malate are transferred from mitochondria to peroxisomes, with aspartate and 2-OG moving in the opposite direction (Dry et al. 1987) (Fig. 10A).

We propose here that the function of UCPs in the transport of amino acids and dicarboxylates makes them clear candidates for co-option into the NAD-ME subtype C_4_ cycle. Consistent with other transporters proposed to be involved in the NAD-ME C_4_ cycle above, *UCP1* shows bundle sheath–preferential expression in the NAD-ME subtype C_4_ species *P. virgatum* (Fig. 10B) and *G. gynandra*^88^ and low expression in NADP-ME subtype C_4_ (Fig. 10B).

### A transport model for NAD-ME C_4_ photosynthesis

The combination of transport activities at the mitochondrial membrane discussed above enables the proposal of a molecular map for mitochondrial dicarboxylate transport in NAD-ME subtype C_4_ photosynthesis (Fig. 10C). Specifically, UCP facilitates the import of aspartate in exchange for glutamate produced by mitochondrial AspAT-mediated transamination of the aspartate and 2-OG, which in turn is imported by DTC in exchange for malate export. Finally, malate must be reimported through DIC in exchange for P_i_ export, driven by the P_i_ gradient generated by P_i_:H^+^ symport through PIC (Fig. 10C). Remarkably, this transport model was first proposed by Furbank et al. (Furbank et al. 1990) before any of the transporters involved had been identified. Their insight was derived from the observation that isolated *P. miliaceum* bundle sheath cells and chloroplasts carry out maximal O_2_ evolution only in the presence of a combination of aspartate, 2-OG, malate, and P_i_ (Furbank et al. 1990). The model was further supported by Taniguchi and Sugiyama after the initial characterization of DTC in a C_4_ plant (Taniguchi and Sugiyama 1997). Now that candidate transporters capable of carrying out these reactions have been identified, it provides the first complete molecular map for transport in NAD-ME C_4_ photosynthesis and will enable molecular dissection of the cycle.

### There are major gaps in our understanding of metabolite transport in C_4_ photosynthesis

Substantial progress has been made in our understanding of the metabolite transporters of the C_4_ cycle. However, there are still several important transport steps for which no known transporters have been shown to provide the required movement. For example, while most C_4_ species appear to have recruited the Na^+^-dependent BASS2 for pyruvate import into the mesophyll chloroplast, the transporter that facilitates the H^+^-dependent pyruvate uptake in the economically important *Arundinelleae* and *Andropogoneae* has yet to be discovered (Aoki et al. 1992). Similarly, the transporters that facilitate malate uptake and pyruvate export in bundle sheath chloroplast of NADP-ME subtype C_4_ species are still unknown. The best evidence to date suggests that malate uptake in the majority of NADP-ME C_4_ species utilize a multi-step valve-like mechanism that is in part facilitated by members of the DCT subfamily (Weissmann et al. 2016). However, the only efficient transport activity so far observed for DCT subfamily transporters is the exchange of 4- and 5-carbon dicarboxylates (e.g. OAA, malate, succinate, fumarate, 2-OG) and amino acids (e.g. aspartate, glutamate) (Day and Hatch 1981; Renné et al. 2003; Taniguchi et al. 2004). Significant dicarboxylate/amino acid unidirectional uptake has not been observed with DCTs from *A. thaliana* or maize (Taniguchi et al. 2002, 2004), and DCT from C_3_ spinach and C_4_  *F. bidentis* were shown not to exchange malate and pyruvate (Renné et al. 2003). Thus, while DCTs are likely to be involved in the transport of malate across the bundle sheath chloroplast envelope, they cannot facilitate the net import of carbon that is required for the C_4_ cycle to function as a carbon-concentrating mechanism. Therefore, other transporters must play a role.

The existence of an as-yet-unidentified aspartate transporter (likely a cation:aspartate symporter) has been proposed to try to explain how malate is imported into the chloroplast (Weissmann et al. 2016). This is consistent with previous observations that malate-dependent pyruvate formation from wild-type maize bundle sheath cells or chloroplasts is stimulated by aspartate (Chapman and Hatch 1979; Jenkins and Boag 1985). It is also plausible that there exists a separate transporter capable of either malate uptake (likely a cation:malate symporter) or simultaneous malate uptake and pyruvate efflux that could provide this function. In the absence of a transporter capable of carrying out malate/pyruvate exchange, pyruvate export is presumably facilitated by a separate pyruvate exporter (likely a cation:pyruvate symporter). However, no such transporter is known in plants or other organisms. Insights may come from the identification of an as-yet-unknown transporter that facilitates pyruvate efflux from mitochondria discussed above (Le et al. 2021).

Advances in methods that could enable high throughput determination of transport activities of candidate transporters identified from transcriptomic data would substantially advance this search, particularly for pyruvate, which is difficult to analyse in assays that require reconstituted liposomes. For example, methods such as fluorescence-detection size-exclusion chromatography-based thermostability assays (Hattori et al. 2012), nano differential scanning fluorimetry (Gao et al. 2020), tryptophan fluorescence quenching (Yammine et al. 2019), or surface plasmon resonance (Patching 2014) could be used to accelerate screening of candidate transporters for their ability to bind to specific substrates. Subsequent transport validation could then be achieved using genetically encoded biosensors (Ho and Frommer 2014) or radioactive in vivo uptake methods prior to kinetic characterization in proteoliposomes.

### There is a pressing need to characterize C_4_ cycle transporters in C_4_ plants

Although many transporters capable of catalyzing the correct transport reactions required for C_4_ photosynthesis have been characterized in C_3_ species, only 1 (maize DCT) has been subject to physiological evaluation in C_4_ species (Weissmann et al. 2016). Thus, direct assessment of the exact role these transporters play in C_4_ photosynthesis is lacking. This creates several problems. Most importantly, the involvement of almost all the transporters proposed to facilitate all C_4_ cycles are currently just hypotheses, with multiple working in opposite directions to those characterised in C_3_ plants or other eukaryotes. This lack of experimental validation in C_4_ plants is likely masking complexities to the transport models and metabolic pathways, leading to misunderstandings concerning the operation of the C_4_ cycle. For example, from initial transcriptomic studies it was assumed that transporters from the DCT family facilitate malate uptake into the chloroplast of NADP-ME species (Chang et al. 2012). However, when these transporters were knocked out, malate was still taken up, albeit at a reduced rate, revealing that malate import into the bundle sheath chloroplast is more complex than first thought (Weissmann et al. 2016). It is likely that other such hidden features of C_4_ photosynthesis will be revealed if other proposed C_4_ cycle transporters are interrogated in C_4_ plants themselves. Continual advances in transformation, DNA synthesis, genomic resources, and genome editing technologies make these experimental investigations more tractable, and these are a clear priority for C_4_ photosynthesis research. However, it may prove challenging to generate transporter loss-of-function lines in C_4_ plants if the resulting plants prove to be nonviable. Moreover, phenotypic evaluation may be complicated by the requirement to grow plants at high CO_2_ concentrations.

## Conclusion

C_4_ photosynthesis is one of the most fascinating examples of complex convergent evolution in eukaryotic biology. Understanding how this convergent trait is manifest holds immense potential to help us understand how complexity evolves, how the world's most productive photosynthetic pathway operates, and to provide a blueprint to enhance photosynthesis, reduce nitrogen requirements, and reduce water use in C_3_ crop plants. The identification of the missing transporters above and a detailed characterization of transporter function in C_4_ plants represent one of the biggest challenges in achieving these aims and is essential to realize the goal of engineering C_4_ photosynthesis into C_3_ plants to improve their yield.

## Data Availability

There are no new data in this work. All data is available in the referenced articles.

## Dive Curated Terms

The following phenotypic, genotypic, and functional terms are of significance to the work described in this paper:

MPC Gramene: AT5G20090
MPC Araport: AT5G20090

NHD1 Gramene: AT3G19490
NHD1 Araport: AT3G19490

PGK Gramene: AT3G12780
PGK Araport: AT3G12780

DCT Gramene: AT5G64280
DCT Araport: AT5G64280

BASS2 Gramene: AT2G26900
BASS2 Araport: AT2G26900

DIC Gramene: AT2G22500
DIC Araport: AT2G22500

OMT1 Gramene: AT5G12860
OMT1 Araport: AT5G12860

TPT Gramene: At5g46110
TPT Araport: At5g46110

PEPC Gramene: Sobic.010G160700
PEPC Araport: Sobic.010G160700

PPDK Gramene: Sobic.009G132900
PPDK Araport: Sobic.009G132900

UCP1 Gramene: AT3G54110
UCP1 Araport: AT3G54110

UCP Gramene: AT5G58970
UCP Araport: AT5G58970

PPT1 Gramene: AT5G33320
PPT1 Araport: AT5G33320
